# Transcriptional Innate Immune Response of the Developing Chicken Embryo to Newcastle Disease Virus Infection

**DOI:** 10.3389/fgene.2018.00061

**Published:** 2018-02-27

**Authors:** Megan A. Schilling, Robab Katani, Sahar Memari, Meredith Cavanaugh, Joram Buza, Jessica Radzio-Basu, Fulgence N. Mpenda, Melissa S. Deist, Susan J. Lamont, Vivek Kapur

**Affiliations:** ^1^Huck Institutes of the Life Sciences, Pennsylvania State University, University Park, PA, United States; ^2^Department of Animal Science, Pennsylvania State University, University Park, PA, United States; ^3^School of Life Sciences and Bio-Engineering, The Nelson Mandela African Institution of Science and Technology, Arusha, Tanzania; ^4^Applied Biological Research Laboratory, Pennsylvania State University, University Park, PA, United States; ^5^Department of Biology, Pennsylvania State University, University Park, PA, United States; ^6^Department of Animal Science, Iowa State University, Ames, IA, United States

**Keywords:** backyard poultry, chicken embryo, Newcastle disease virus, innate immune response, transcriptional response

## Abstract

Traditional approaches to assess the immune response of chickens to infection are through animal trials, which are expensive, require enhanced biosecurity, compromise welfare, and are frequently influenced by confounding variables. Since the chicken embryo becomes immunocompetent prior to hatch, we here characterized the transcriptional response of selected innate immune genes to Newcastle disease virus (NDV) infection in chicken embryos at days 10, 14, and 18 of embryonic development. The results suggest that the innate immune response 72 h after challenge of 18-day chicken embryo is both consistent and robust. The expression of CCL5, Mx1, and TLR3 in lung tissues of NDV challenged chicken embryos from the outbred Kuroiler and Tanzanian local ecotype lines showed that their expression was several orders of magnitude higher in the Kuroiler than in the local ecotypes. Next, the expression patterns of three additional innate-immunity related genes, IL-8, IRF-1, and STAT1, were examined in the highly congenic Fayoumi (M5.1 and M15.2) and Leghorn (Ghs6 and Ghs13) sublines that differ only at the microchromosome bearing the major histocompatibility locus. The results show that the Ghs13 Leghorn subline had a consistently higher expression of all genes except IL-8 and expression seemed to be subline-dependent rather than breed-dependent, suggesting that the innate immune response of chicken embryos to NDV infection may be genetically controlled by the MHC-locus. Taken together, the results suggest that the chicken embryo may represent a promising model to studying the patterns and sources of variation of the avian innate immune response to infection with NDV and related pathogens.

## Introduction

Newcastle disease virus (NDV) is one of the most important poultry pathogens worldwide, with over eighty countries in North and South America, Europe, Asia, and Africa reporting outbreaks each year (Diel et al., 2012). NDV infections manifest through a wide range of strain dependent symptoms including those within the respiratory system (coughing, sneezing, and wheezing), the nervous system (such as twisted neck, tremors, and paralysis), and the reproductive system (loss in egg production). Mortality rates may reach as high as 100% in unvaccinated flocks (Ashraf and Shah, 2014). Unsurprisingly then, NDV infections are responsible for considerable economic losses to poultry production in both developed and developing countries. For instance, the 2002–2003 NDV outbreak in California resulted in the destruction of 3 million birds and financial losses of over $160 million (Diel et al., 2012).

Differing levels of susceptibility to NDV have been reported among commercial breeds, as well as between commercial poultry and local ecotypes; however, the mechanisms contributing to these differences remain unknown (Zhou and Lamont, 1999; Minga et al., 2004; Deist et al., 2017b). For instance, studies report that the highly inbred Fayoumi lines are less susceptible to NDV infection (and other infections) than single comb, highly inbred White Leghorn chickens (Zhou and Lamont, 1999). Similarly, backyard chickens are generally considered less susceptible to NDV and other infections than commercial chickens that are bred for high productivity. This reduced susceptibility is presumably due to their pre-sensitization with a higher frequency of natural exposure to pathogens in the scavenging environment, as well as through natural selection for hardiness to higher levels of pathogen exposure. However, considerable variation in response to NDV infection has been noted both between and within ecotypes commonly found in backyard settings (Minga et al., 2004). Another breed of chickens hypothesized to be less susceptible to disease is the Kuroiler, a dual-purpose chicken, first bred for improving both meat and egg production of backyard poultry in India. The Kuroiler, which has multi-colored feathers to help with camouflage in the wild, and thrives in backyard or scavenging environments has recently been introduced in East Africa (including Tanzania) with reports suggesting that it can coexist with, and out-produce the local chickens in Uganda and Kenya (Dessie and Getachew, 2016).

Although differences in susceptibility are observed between these breeds, the underlying immune mechanisms contributing to these differences remain unknown. With the widespread use of NDV vaccines, studies of immunity have focused on antibody production and cell-mediated responses more so than innate immunity (Ahmed et al., 2007; Kapczynski et al., 2013). One study demonstrated a rapid and robust innate response shortly after virulent NDV infection using a microarray analysis of chicken spleen tissue (Rue et al., 2011). However, the level of susceptibility to NDV was not examined in the study, hence, whether the innate immune response plays a role in enhancing immunity to NDV in poultry, and particularly in backyard poultry, is presently not understood.

Current techniques to evaluate the immune response and disease susceptibility in chickens through challenge of live birds are expensive and difficult to interpret due to confounding factors including age, natural exposure to infectious agents, the normal microflora, variability in dosing animals through natural routes of exposure, nutritional status, as well as exposure to other environmental stressors. In contrast, the chicken embryo, enclosed in the protective environment of the shell, is not only considerably cheaper, but also may be influenced by confounding environmental factors that may impact the results of challenge studies in hatched chicks.

It has been well described that the developing chicken embryo is able to produce an immune response to a pathogen prior to hatch, a feature that is widely exploited for modern large-scale poultry production with the routine administration of *in ovo* vaccination for multiple pathogens, including Marek’s Disease (MD) and Infectious Bursal Disease (IBD) (Sharma and Burmester, 1984; Stone et al., 1997; Seal et al., 2000). Embryonic development occurs over 21 days and by the 10th day, the first signs of the immune system are observed. On days 11 and 12, T cells and B cells are developed, respectively, with B cell differentiation occurring after the 15th day of development. By the 18th day of embryonic development, the chicken embryo is immunocompetent and is capable of producing both an innate and adaptive response to pathogen (Davison, 2003; Ribatti, 2010; Mississippi State University Extension, 2017). Importantly, immune responses have been shown to be comparable in birds vaccinated *in ovo* or at later time points post-hatch (Sharma and Burmester, 1984; Stone et al., 1997; Gagic et al., 1999; Sharma, 1999; Steel et al., 2008). For example, vaccination with an inactivated oil emulsion NDV and Avian Influenza Virus (AIV) vaccine on the 18th day of embryonic development resulted in high seroconversion rates and antibody titers post hatch (Stone et al., 1997). An MD *in ovo* vaccine was also able to generate a four times greater level of protection than post hatch vaccination (Sharma and Burmester, 1984). Even though *in ovo* vaccination has now been applied for several decades, the mechanisms of induction of protective immunity in the chicken embryo remain poorly understood.

To begin to address this knowledge gap, we here transcriptionally profiled the innate immune response of the chicken embryo to NDV infection in both highly inbred and outbred lines. Our studies begin to demonstrate the use of the chicken embryo as a tool to examine the immune response to NDV since signatures of a consistent, breed-dependent innate immune response post NDV infection are present.

## Materials and Methods

### Ethics Statement and Animal Use

Animal use protocols were approved by the Pennsylvania State University IACUC committee (protocol numbers 46395 and 47175). Specific pathogen free (SPF) eggs from White Leghorn chickens were sourced through Charles River Laboratories International, Inc. (North Franklin, CT, United States). Tanzanian local ecotype and Kuroiler hatching eggs were sourced from Urio Cross and Pure Breeding LTD, a local farm, in Tanzania (Tengeru, Arusha, Tanzania). Embryonated eggs from two well-defined, inbred Leghorn sublines, Ghs6 and Ghs13, as well as two inbred Fayoumi sublines, M5.1 and M15.2, from Iowa State University Poultry Farm (Ames, IA, United States) were also included in this study. Eggs were incubated (37.5°C, 55% humidity, rotating hourly) and only temporarily removed from incubation to candle for viability and perform inoculations with virus.

### Virus

The lentogenic LaSota strain of NDV was kindly provided by Dr. Siba Samal at the University of Maryland, College of Veterinary Medicine (College Park, MD, United States). Titration of the virus was performed with the final titer of undiluted viral suspension of 10^7^ 50% egg infectious dose (EID_50_)/mL. The viral suspension was stored at -80°C until further use.

### Embryonated Chicken Egg Infection with NDV and Chicken Embryo Tissue Harvest

Embryonated eggs were inoculated at days 10, 14, or 18 of embryonic development, respectively, with 0.1 mL of the viral suspension directly deposited into the allantoic fluid. Eggs were sealed with an adhesive glue and placed back in incubators until death or removal for tissue harvest. Controls (uninfected eggs) were treated similarly with inoculation of 0.1 mL of phosphate buffered saline (PBS). Eggs were candled daily once infected and embryo (including dead embryo at that time point) were randomly selected for tissue harvest 24, 48, and 72 h post infection (hpi). The embryo selected in the group infected at 18 days and harvested 72 hpi were as close to hatch as possible without allowing the chick to hatch. Prior to harvest, the eggs were placed at 4°C for 3–4 h to avoid opening eggs with viable embryos, the harvested embryo was extracted and rinsed three times in 1X PBS before selected tissues (based on size of the embryo) were harvested (Supplementary Table S1). Tissues harvested at the 10-day infection included two sections, the body and head, as the chicken embryo was too small and underdeveloped to obtain individual tissue samples. After the 14-day infection, the spleen, heart, and liver were harvested, and after 18-day infection, the spleen, heart, liver, and lung tissues were harvested. In the experiment using the SPF White Leghorn eggs, tissues from three infected and three control embryo were harvested at each time point; for the Kuroiler and Local ecotype embryos, eight infected and eight control embryos were harvested; and for the Leghorn and Fayoumi sublines, six infected and six control were harvested in the Ghs6, Ghs13, and M5.1 and eight infected and six control embryos were harvested for M15.2. The tissues were immediately stored at -80°C prior to further use.

### RNA Extraction

RNA from all tissues was extracted using the RNeasy Plus Kit (QIAGEN Inc., Germantown, MD, United States) following the recommended protocol once tissues were homogenized. Tissue homogenization was performed using a Mini-Beadbeater-96 (Biospec Products, Bartlesville, OK, United States) for 1 min. Each tube placed in the beat beater contained 100 mg of respective tissue, 600 μL of RLT Lysis buffer (provided in the RNeasy kit), and 10–15 1.5 mm silica beads (Biospec Products, Bartlesville, OK, United States). After homogenization, 600 μL of lysate was added to a clean microcentrifuge tube and centrifuged at 10,000 rpm for 3 min to remove any debris. The supernatant was transferred to the gDNA eliminator column (provided in the RNeasy kit) and the recommended protocol was followed from this point forward.

### cDNA Synthesis

cDNA synthesis was performed immediately after RNA extraction using the RT^2^ First Strand Kit (QIAGEN Inc., Germantown, MD, United States) for the samples used with the RT^2^ Profiler Array (one control and one infected embryo at 18 days and harvested 72 h post infection) and the High Capacity cDNA Reverse Transcription Kit (Applied Biosystems, Carlsbad, CA, United States) for all other samples at all other time points. Manufacturer protocols were followed using 2 μg of each respective RNA sample. cDNA was stored at -20°C.

### Transcriptional Profiling of Innate and Adaptive Immune Responses

The RT^2^ Profiler Array Chicken Innate and Adaptive Immune Responses (QIAGEN Inc., Germantown, MD, United States) was used as an initial screen of the immune response of embryo to NDV infection. The array contains a panel of 84 immune-related genes from the innate (Pattern Recognition Receptors, Cytokines, Other), adaptive (Th1, Th2, Th17, and Treg markers, T Cell Activation, Cytokines, Other), humoral, and inflammatory responses, as well as the defense response to bacteria and virus (QIAGEN, 2014). All recommended kits and procedures were followed (QIAGEN Inc., Germantown, MD, United States). A master mix was prepared using the RT^2^ qPCR Master Mix and cDNA according to manufacturer protocols and added to each well of the 96 well plate, provided by QIAGEN Inc. qPCR was then performed using an Applied Biosystems StepOne Plus real-time PCR instrument per recommended protocols.

At the Pennsylvania State University, the PowerUp SYBR Green Master Mix (Applied Biosystems, Carlsbad, CA, United States) with the StepOne Plus System (Applied Biosystems, Carlsbad, CA, United States) was used to analyze the SPF White Leghorn, inbred Leghorn, and inbred Fayoumi samples. At the Nelson Mandela African Institution of Science and Technology, the QuantiNova SYBR Green RT-PCR Kit (QIAGEN Inc., Germantown, MD, United States) with the QuantStudio 6 Flex Real-Time PCR System (Applied Biosystems, Carlsbad, CA, United States) was used to analyze the Kuroiler and local ecotype samples. The recommended manufacturer’s protocols were followed for both kits. The differences in protocols were due to availability of reagents and machines at NM-AIST. The QuantiNova kit was recommended as most stable for shipment to Tanzania. The primers used for analysis can be found in Supplementary Table S2.

### Data Analysis

Gene expression was analyzed using the ΔΔCt method comparing infected ΔCt values (normalized with B-actin) with the average of negative ΔCt values (normalized with B-actin) for each gene studied. The figures were generated in R^1^ using log2 fold change expression data obtained from the ΔΔCt method for gene expression analysis. Statistical analysis performed between the Kuroiler and local ecotypes was performed in R using the Student’s *t*-test and between the Leghorn and Fayoumi sublines using the non-parametric pairwise *t*-test (Kruskal–Wallis test) in (R Core Team, 2016).

Gene network analysis was performed using the Ingenuity Pathway Analysis (IPA) software (QIAGEN Inc., Germantown, MD, United States^2^) (QIAGEN, 2014). The data was uploaded to IPA and Core analysis was performed to generate the network in **Figure 2A**. From this the genes with the most gene–gene interactions and highest fold changes were selected (**Figure 2B**). The dataset was filtered for these six genes and the top diseases and biofunctions, in particular related to infectious disease and relevant to this study, were selected and the network in **Figure 2C** was generated. The same networking was performed for the four sublines (**Figure 4**).

## Results

Studies were performed to define the point during embryonic development when the chicken embryo is capable of producing a robust and consistent immune response to NDV, as well as to identify specific immune genes with the greatest differential expression post infection. Developing chicken embryos of various ages were infected with the LaSota strain of NDV via the allantoic fluid and tissues were harvested 24, 48, and 72 h post infection (hpi) (Supplementary Table S1).

The comparative transcriptional profile of SPF White Leghorn chicken embryos (one control and one infected at 18 days, harvested 72 hpi) was determined using the Chicken Innate and Adaptive Immune Responses RT^2^ Profiler Array (QIAGEN Inc., Germantown, MD, United States) as an initial screen to select for immune genes in the embryo that are differentially expressed during infection since studies have not been performed previously. The upregulated genes range from 1-fold (IRF6 gene) to 754-fold (Mx1 gene) increases, and the downregulated genes range from -1-fold (IL1R1 gene) to -11-fold (CCR4 gene) decreases (Supplementary Table S3). Three of the genes with the highest increase in expression were selected for a more comprehensive analysis of the immune response in the embryo, C-C Motif Chemokine Ligand 5 (CCL5), MX Dynamin Like GTPase 1 (Mx1), and Toll-like Receptor 3 (TLR3). These three genes are also known to play a role in the innate immune response, in particular the immune response to viral infection (QIAGEN, 2014). This data is publically available at https://doi.org/10.18113/D3H952.

Once the three genes above were selected for further studies, the other collected embryo tissues from the SPF White Leghorns (three infected embryos and three control embryos per time point) were examined for the expression of these genes throughout the immune development of the chicken embryo (Supplementary Figure S1 and Table S1). Expression of all three immune genes was increased in the infected lung tissues at 18 days of embryonic development 72 hpi with considerably lower variation in expression than other infection time points (Supplementary Figure S1). Additionally, the Mx1 gene had a greater fold increase in the lung tissues as compared to CCL5 and TLR3 (average fold changes of 254, 4.2, and 5.6, respectively). Infection of the chicken embryo at 18 days, in particular with harvest of tissues 72 hpi, was deemed the most suitable time point for examining the immune response of the chicken embryo to NDV infection.

**FIGURE 1:**
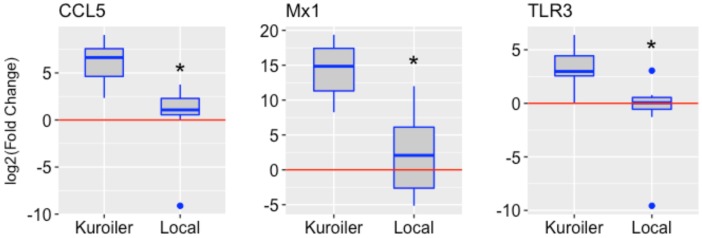
Expression of CCL5, Mx1, and TLR3 in NDV-infected embryos from the Kuroiler and local ecotypes in Tanzania. Differences in the log2 of the fold change are shown in the boxplots. The red line signifies the cutoff between upregulation and downregulation of the genes. Gene expression is significantly different between the Kuroiler and Local ecotypes (*n* = 8 infected, 8 control per line) for all of the genes studied (^∗^*p*-values < 0.001).

After determining a suitable time point to study chicken embryo immune gene expression, the analysis was broadened to include the Kuroiler and local ecotypes in Tanzania. The same three genes, CCL5, Mx1, and TLR3, were examined in the lung tissue of the Kuroiler and local ecotype embryos infected at 18 days of embryonic development and harvested 72 hpi (eight infected embryos and eight control embryos per line). Striking differences in expression of immune genes were observed between the Kuroiler and local ecotypes. The Kuroiler consistently expressed CCL5, Mx1, and TLR3 several orders of magnitude greater (164-, 19,816-, and 21.8-fold increase, respectively) than the local ecotype embryos (4.2-, 4.7-, and 1.8-fold increase, respectively) (**Figure 1**; data for individual replicates^3^).

**FIGURE 2:**
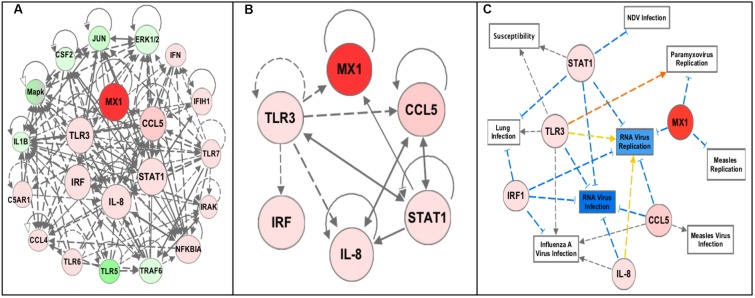
Pathway analysis of the RT^2^ Profiler Array gene expression data using Ingenuity Pathway Analysis (IPA) software. The red colored circles represent relative increases in expression of that gene in the infected chicken embryo lung tissues versus control and green circles represent downregulation. Dashed lines indicate direct relationships and solid lines indicate indirect relationships. The blue lines represent inhibition, orange represents activation, and yellow represents inconsistent findings with downstream molecules. **(A)** Networks associated with diseases and functions of the differentially expressed genes from the profiler array (Infectious Diseases, Cell-mediated Immune Responses, Lymphoid Tissue Development, and Organismal Injury) were mapped together. **(B)** The six highly upregulated and interconnected genes (Mx1, CCL5, TLR3, STAT1, IL-8, and IRF1) from the array were selected for future analysis. **(C)** Filtering of the data to include only these six genes revealed diseases and functions relevant to NDV infection, including NDV infection, Paramyxovirus replication, RNA virus infection and replication, susceptibility to infection, lung infection, Measles virus infection and replication, and Influenza A virus infection (all *p*-values < 0.0001).

Pathway analysis using the Ingenuity Pathway Analysis (IPA) software (QIAGEN Inc., Germantown, MD, United States^2^) was performed using the RT^2^ Profiler Array data (expression fold change cutoff = 2) to determine important pathways and networks in the response to NDV in the chicken embryo (QIAGEN, 2014). The top canonical pathways included the role of pattern recognition receptors in recognition of bacteria and virus, iNOS Signaling, communication between innate and adaptive immune cells, and Toll-like receptor signaling (each pathway – *p*-values < 0.0001). The top regulator effector network was the antiviral response. The identified associated network diseases and functions including infectious disease, the cell-mediated immune response, and organismal injury, were selected based on relevance to the immune response and merged to generate a network map of the gene–gene interactions from the RT^2^ Profiler Array, removing genes that were not included in the array (**Figure 2A**). From this, an additional three genes, Interleukin-8 (IL-8), Interferon Regulatory Factor 1 (IRF1) and Signal Transducer and Activator of Transcription 1 (STAT1), were selected for future analysis since they were all highly increased in the infected chicken embryo as well as being interconnected in immune response networks (**Figure 2B**). Core analysis was performed with filtering for these genes to determine important networks of relevance to this study with NDV (**Figure 2C**). Remarkably, the diseases and disorders associated with the expression of these six genes include NDV infection, RNA virus infection and replication, Paramyxovirus replication, lung infection, Measles virus infection and replication (a paramyxovirus), and Influenza A virus infection (a RNA virus of the respiratory tract), and susceptibility (*p*-values < 0.0001 for all diseases and disorders listed) (QIAGEN, 2014). However, we note that even though IPA analysis has been previously applied to understand patterns of gene expression in chickens, the pathways have been validated primarily using human/mouse/rat data, and it is also recommended to use at least 200 genes for pathways analyses, and hence, the results should be independently replicated (Krämer et al., 2014).

**FIGURE 3:**
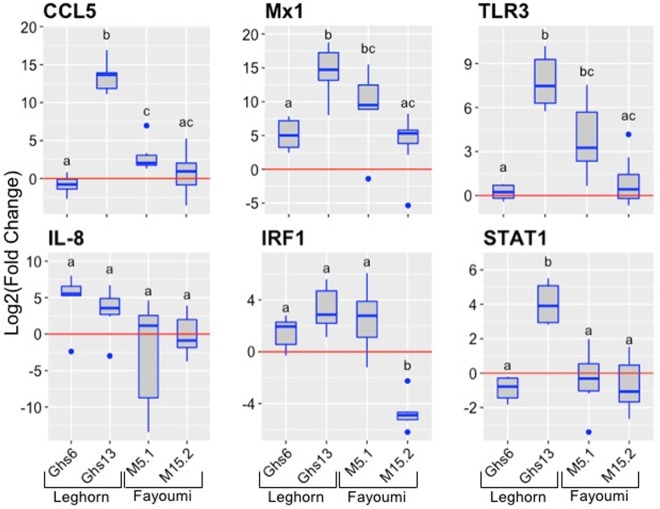
Expression of immune genes in the Leghorn (Ghs6 and Ghs13) and Fayoumi (M5.1 and M15.2) sublines. The six immune genes were examined in the lung tissues of each subline (Ghs6, Ghs13, M5.1 – *n* = 6 infected and 6 controls per subline; M15.2 – *n* = 8 infected and 6 control). The red line signifies the cutoff between upregulation and downregulation of the genes. Statistical analysis was performed according to the materials and methods data analysis and significant *p*-values noted by the superscripts (*p*-value < 0.05) can be found in Supplementary Table S4.

Next, the immune response profiles of 18-day NDV-infected embryos of the well-defined, highly congenic Fayoumi (M5.1 and M15.2) and Leghorn (Ghs6 and Ghs13) sublines were characterized (six infected embryos and six control embryos per subline – Ghs6, Ghs13, M5.1; eight infected and six control for the M15.2 subline). The congenic Fayoumi and Leghorn lines are highly inbred commercial poultry with inbreeding coefficients of 0.99, differing within each line only at the microchromosome bearing the MHC (Zhou and Lamont, 1999; Deist et al., 2017b). The Fayoumi was described as less susceptible to infectious disease than the Leghorn (Zhou and Lamont, 1999; Cheeseman, 2007). One study showed the Fayoumi line (M15.2) to be less susceptible to NDV than the Leghorn line (Ghs6) based on viral load post-challenge in ocular secretions at 6 (but not 2) days post infection (Hermann et al., 2016; Deist et al., 2017b), and have also identified differentially expressed genes in the trachea and lung transcriptomes of these two highly inbred chicken lines (Deist et al., 2017a,b). The results of our current investigation show striking differential expression of select genes in a subline-dependent manner with Ghs13 having consistently higher expression of all genes except IL-8 (**Figure 3**; data for individual replicates^4^). Importantly, the results show that the gene expression differences seem to be subline-dependent even more so than breed dependent.

**FIGURE 4:**
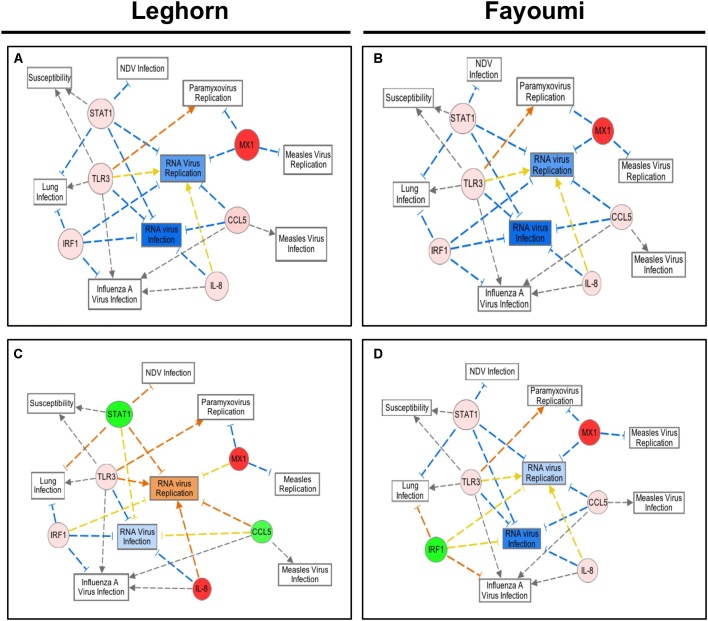
Network Analysis of the six selected immune genes in the Leghorn and Fayoumi sublines using IPA software. The data used for this analysis is from the gene expression data from each Fayoumi and Leghorn subline in **Figure 3**. The red colored circles represent relative increases in expression of that gene in the infected chicken embryo lung tissues versus control and green circles represent downregulation. Dashed lines indicate direct relationships and solid lines indicate indirect relationships. The blue lines represent inhibition, orange represents activation, and yellow represents inconsistent findings with downstream molecules. Ghs13 **(A)** and M5.1 **(B)** the highest responders have similar patterns in the network analysis, and Ghs6 **(C)** and M15.2 **(D)** show deviations from this network due to the downregulation of some genes.

Similar analysis was performed, as in **Figure 2C**, with the average fold expression differences between challenged and control lung tissue in the inbred Leghorn and inbred Fayoumi sublines using IPA (QIAGEN Inc., Germantown, MD, United States^5^). Interestingly, the high responders, Ghs13 and M5.2 (**Figures 4A,B**, respectively), mimic similar patterns as the RT^2^ Profiler Array analysis (**Figure 3**). The low responders, Ghs6 and M15.2 (**Figures 4C,D**, respectively), differ especially due to the downregulation of one or more of these genes, STAT1, CCL5, and IRF1, was predicted to increase viral replication and infection in the Ghs6 and M15.2 sublines (QIAGEN, 2014).

## Discussion

Until now, most studies performed to examine the immune response of chickens and other avian species have been through expensive and laborious *in vivo* experiments or through *in vitro* cell culture experiments lacking host–pathogen interactions. The animal studies, requiring enhanced biosecurity facilities, are influenced by multiple confounding factors producing results that are sometimes difficult to interpret. Therefore, despite current work to examine the chicken immune response to NDV, a knowledge gap remains in the mechanisms of the immune response and the effects they have on susceptibility of chickens to infection. In the present study, we utilized the developing chicken embryo as a controlled and inexpensive approach to evaluate the innate immune mechanisms in response to NDV infection in chickens. Through these studies we have found evidence that the chicken embryo is capable of producing a robust signature of the immune response, in particular the innate immune response, to NDV infection, at least after day 10 of embryonic development. In particular, the suitability of the chicken embryo for immune investigation was confirmed through gene expression studies where variation in gene expression was greatly reduced at 18-day infection as compared to earlier infection time points (Supplementary Figure S1). Since the chicken embryo is immunocompetent and the respiratory system almost fully developed a few days prior to hatch, examining the response of the chicken embryo to respiratory viruses such as NDV may be possible without the introduction of other confounding variables post-hatch.

Using this more controlled approach to examine the chicken immune response, we were able to demonstrate that the innate immune response to NDV in the chicken embryo appeared to be breed- (**Figure 1**) and/or subline-dependent (**Figure 3**), with the possibility of having a relation to the MHC type demonstrated by differences in the inbred Fayoumi and inbred Leghorn sublines, which only differ in the MHC-bearing chromosome. If the gene expression is, in fact, MHC type dependent, this could explain some of the large within breed variation in expression demonstrated by the Tanzanian local ecotypes and the Kuroiler. These differences in the transcriptional response to NDV infection also demonstrate a possible relation between the innate immune gene expression and level of susceptibility of a particular line, since previous reports demonstrate the Ghs6 Leghorn subline and M15.2 Fayoumi subline differ in the level of susceptibility to NDV (Deist et al., 2017b). This study also showed IL-8 and Mx1 to be differentially expressed between the two sublines studied (Deist et al., 2017b). A more recent investigation of differential gene expression in the lung transcriptome of these same chicken lines in response to NDV infection also, demonstrated an activation of the IL-8 pathway in the resistant Fayoumi line, M15.2 (Deist et al., 2017a). While these previous studies used RNA-Seq based approaches and longer time-points (days rather than hours) post-infection and performed these studies in hatched chicks and are hence not directly comparable, the similar patterns of expression of at least some of the loci observed in the chick embryo model are encouraging, and need to be confirmed in future investigations.

Multiple other studies have each discovered at least one of the six genes (Mx1, CCL5, TLR3, IL-8, IRF1, and STAT1) studied here with elevated expression levels in the chicken particularly in response to NDV, AIV, and IBV demonstrating the key role of these innate immune genes in the chicken’s response to pathogen (Heidari et al., 2010; Rue et al., 2011; Matulova et al., 2013; Cheng et al., 2014; Kang et al., 2016; Ranaware et al., 2016; Deist et al., 2017b). Although the network analysis was only performed with small numbers of genes, relevant pathways to our study were generated. Most surprising were the pathways, top diseases, and disorders associated with the genes including NDV infection, paramyxovirus replication, and lung infection since they are closely associated with the study of NDV infection in the chicken embryo lung directly. Another significant function of relevance to this study was susceptibility to infection. Since determining the feasibility of using the chicken embryo as a new approach to explore the immune response and susceptibility to NDV infection is an ultimate goal of our project, revealing a significant involvement of STAT1 and TLR3 in this response is promising for future studies. Other diseases and disorders include RNA virus infection and replication (NDV is an RNA virus), Measles virus infection and replication (a paramyxovirus, like NDV), and Influenza A virus infection (an RNA virus of the respiratory tract) (QIAGEN, 2014). In most cases, TLR3, STAT1, and IRF1 are involved in signaling pathways that lead to stimulation of the end targets, CCL5, Mx1, and IL-8, which then go on to stimulate other immune related cells or pathways (QIAGEN, 2014). Multiple studies have recognized the Type-I Interferon (IFN) inducible response, protecting cells against invading viral pathogens, as important in the innate immune response in chickens, and the six genes examined in this study are involved in that response (Xing et al., 2008; Schoggins et al., 2011; Ranaware et al., 2016). The six genes studied here have been shown to be important in the chicken immune response to viral pathogens, whether it is NDV, AIV, or IBV (Ko et al., 2002; Benfield et al., 2008; Sartika et al., 2011; Cong et al., 2013; Barjesteh et al., 2014; Cheng et al., 2014; Dou et al., 2014; Fulton et al., 2014; Ranaware et al., 2016; Deist et al., 2017a,b), which demonstrates how the chicken embryo immune response starts to mimic that of hatched chickens, validating the use of the chicken embryo for future studies of the immune response to avian pathogens.

The innate immune response is a complex and interconnected network that is dependent on many factors. Although the six genes examine in this study are involved in the innate immune response and there are direct interactions between these genes, further validation of the response, including a larger set of innate immune genes, is needed in order to obtain a better understanding of the innate immune mechanisms influencing the chicken embryo and chicken’s response to NDV infection. It is interesting to note the differences seen in the pathway analysis from the Leghorn and Fayoumi sublines and a possible role for these genes in determining the level of susceptibility of a particular line to NDV infection. Most interestingly, the Ghs6 subline, previously found to be susceptible to NDV, differs from the other sublines pathway analysis by activating RNA virus replication rather than inhibiting it (QIAGEN, 2014; Deist et al., 2017b). Since only the Ghs6 and M15.2 sublines have been previously characterized for the level of susceptibility to NDV, expanding research in this area to include the other two sublines may provide a better insight into the use of the chicken embryo immune response as a tool to screen for the level of susceptibility to NDV of a particular line, and the role of the MHC complex. It is especially intriguing since the high responders, Ghs6 and M5.1, differ in the network analysis from the low responder, Ghs13. A more comprehensive study of the innate immune response of the different chicken lines, including a larger set of genes and larger sample sizes, and correlating these responses to the innate immune response and level of susceptibility of hatched chicks, using phenotypic characteristics such as viral load, mean death time, viral shedding, and antibody titers post NDV infection, will help provide insight into innate immune mechanisms of susceptibility to NDV. Other important future studies include assessing whether similar patterns in response using more pathogenic field strains which is especially important in regards to the response in backyard poultry.

Newcastle disease virus can be particularly devastating for farmers, especially smallholder farmers in Sub-Saharan Africa, due to high mortality rates in unvaccinated flocks. Uncovering innate immune mechanisms related to the level of susceptibility to NDV would have major impacts on productivity for these farmers by informing breeding strategies to produce more robust chickens. We note that the potential use of the chicken embryo as a model provides a framework for future studies of the development of the innate immune response of chickens to NDV (and other pathogens) allowing for screening of large numbers of birds to uncover genetic markers for both disease resistance/susceptibility and productivity traits, such as egg or meat production.

## Author Contributions

MS, VK, JB, and FM designed the study. MS, SM, and MC performed the laboratory experiments. MS, RK, SM, and MC performed the data analysis. MS wrote the manuscript. RK, JR-B, MD, and SL critically revised the content. All authors read and approved the final manuscript.

## Conflict of Interest Statement

The authors declare that the research was conducted in the absence of any commercial or financial relationships that could be construed as a potential conflict of interest.

## Footnotes

1 https://www.r-project.org/

2 https://targetexplorer.ingenuity.com/

3 https://doi.org/10.18113/D3H952

4 https://doi.org/10.18113/D3H952

5 https://targetexplorer.ingenuity.com/
