# Supplementary material for: Transcriptional Innate Immune Response of the Developing Chicken Embryo to Newcastle Disease Virus Infection
Source: Front Genet. 2018 Feb 27;9:61. doi: 10.3389/fgene.2018.00061 (PMC5835104; doi:10.3389/fgene.2018.00061)
Supplement: Supplementary file 1 [file Data_Sheet_1.docx]

Supplementary Material

**Transcriptional innate immune response of the developing chicken embryo to Newcastle disease virus infection**

Megan Schilling^123^, Robab Katani^12^, Sahar Memari^4^, Meredith Cavanaugh^4^, Joram Buza^3^, Jessica Radzio-Basu^1^, Fulgence N. Mpenda^3^, Melissa S. Deist^5^, Susan J. Lamont^5^, Vivek Kapur^123^*

*** Correspondence:** Vivek Kapur vkapur@psu.edu

#
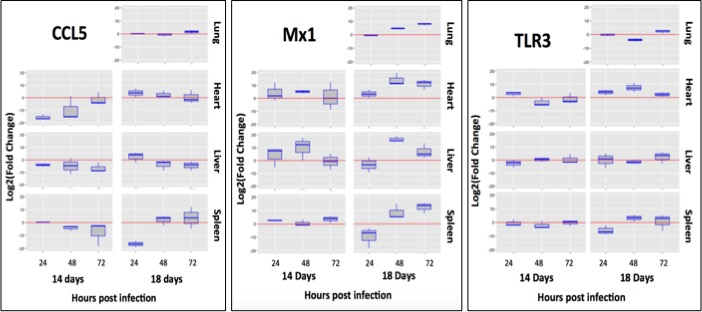
Supplementary Figures and Tables

## Supplementary Figures

**Supplementary Figure 1.** **Gene expression in NDV-infected SPF White Leghorn embryonic tissues.** The three genes (Mx1, CCL5, and TLR3) with the highest fold expression increases were examined through chicken embryonic immune development in different tissues (n = 3 infected and 3 control at each time point). The red line signifies the cutoff between upregulation and downregulation of the genes. The y-axis represents the log2 of the fold change values. Data is shown for the tissues harvested post infection at 14 and 18 days, the data for 10 days is not shown due to high variability and unavailability of different tissues.

## Supplementary Tables

| **Supplementary Table 1. Stratification of Tissue Samples Harvested from Chicken Embryos** | |
| --- | --- |
| **Day of Infection** | **Tissues Harvested** |
| 10 | Head, Body |
| 14 | Heart, Liver, Spleen |
| 18 | Heart, Liver, Spleen, Lung |

**Supplementary Table 1. Stratification of Tissue Samples Harvested from Chicken Embryo.**

| **Supplementary Table 2. RT2 Profiler Array Gene Expression Data** | |
| --- | --- |
| **Gene** | **Fold Change** |
| MX1 | 745.09 |
| CCL5 | 185.85 |
| CCL4 | 65.81 |
| TLR3 | 56.64 |
| STAT1 | 21.45 |
| IL8 | 16.3 |
| IFIH1 | 14.65 |
| IRF1 | 7.39 |
| IRF7 | 7.32 |
| TLR15 | 7.27 |
| MYD88 | 6.56 |
| IFNB | 4.76 |
| TLR2-2 | 4.7 |
| LOC100859040 | 3.85 |
| LITAF | 3.15 |
| NOD1 | 3.11 |
| IFNG | 2.65 |
| C5AR1 | 2.55 |
| LTF | 2.53 |
| IFNAR1 | 2.46 |
| NLRP3 | 2.25 |
| LY96 | 2.19 |
| PTGS2 | 2.04 |
| IL6 | 1.74 |
| IL18 | 1.68 |
| CD40 | 1.6 |
| TLR6 | 1.58 |
| CASP1 | 1.56 |
| FAS | 1.56 |
| MBL2 | 1.56 |
| NFKBIA | 1.49 |
| NFKB1 | 1.43 |
| CD40LG | 1.4 |
| FASLG | 1.38 |
| CD80 | 1.27 |
| STAT3 | 1.19 |
| TLR7 | 1.14 |
| IRAK4 | 1.07 |
| IFNGR1 | 1.06 |
| STAT4 | 1.05 |
| CASP8 | 1.02 |
| IRF6 | 1.01 |
| IL1R1 | -1.01 |
| IL4 | -1.02 |
| CSF2 | -1.04 |
| CD8A | -1.05 |
| TLR21 | -1.1 |
| CD86 | -1.12 |
| IL15 | -1.14 |
| MAPK1 | -1.19 |
| LYZ | -1.2 |
| TICAM1 | -1.21 |
| IL5 | -1.27 |
| TRAF6 | -1.27 |
| JAK2 | -1.32 |
| IL1B | -1.35 |
| LOC430113 | -1.38 |
| CCR5 | -1.4 |
| C3 | -1.46 |
| TLR4 | -1.56 |
| SLC11A1 | -1.57 |
| CAMP | -1.59 |
| CXCL12 | -1.62 |
| ITGB2 | -1.64 |
| CXCR4 | -1.69 |
| GATA3 | -1.69 |
| LOC100859196 | -1.7 |
| IL13 | -1.86 |
| IL2 | -1.86 |
| IL10 | -1.89 |
| MAPK14 | -2 |
| RAG1 | -2.17 |
| MPO | -2.26 |
| JUN | -2.38 |
| CD4 | -2.51 |
| CCR8 | -3.16 |
| IFNA3 | -3.48 |
| CD28 | -3.88 |
| CCR6 | -4.03 |
| CRP | -4.06 |
| MAPK8 | -4.28 |
| TLR5 | -4.85 |
| CD14 | -5.11 |
| CCR4 | -10.46 |

**Supplementary Table 2. RT2 Profiler Array Gene Expression Data.**

| **Supplementary Table 3. Primers used for RT-PCR** | |
| --- | --- |
| **Gene** | **Primers** |
| CCL5 | F-GTTTGGGGCTGATACAACCG |
|  | R-CCTTCACATGATTCTGGGGCA |
| Mx1 | F-CCACAGGAGAAAGGACGCTT |
|  | R-TCTGAGTGGGATGACCTCGT |
| TLR3 | F-TGCTTGGTTTGCTAGTTGGC |
|  | R-CCGTGATATTTAGGCGGGGT |
| IL-8 | F-CAGGTGACACCCGGAAGAAA |
|  | R-CTGAACGTGCCTGAGCCATA |
| STAT1 | F-CCCAAAGGACCTCACAGTCA |
|  | R-TTACTTGATGAAGGCGCCCG |
| IRF1 | F-CAGCACGTTTGGCTACAAGG |
|  | R-TGGTCCATCATGCGGAACTC |
| B-actin | F-GCGCAAGTACTCTGTCTGGA |
|  | R-TTCATCGTACTCCTGCTTGC |

**Supplementary Table 3. Primers used for RT-PCR.**

| **Supplementary Table 4. P-values from Fig 3 - Expression of innate immune genes in the Fayoumi and Leghorn sublines** | | | |
| --- | --- | --- | --- |
| **Target** | **Subline** | | **P-value** |
| CCL5 | Ghs6 | Ghs13 | 0.011 |
| CCL5 | Ghs6 | M5.1 | 0.011 |
| CCL5 | Ghs13 | M5.1 | 0.011 |
| CCL5 | Ghs13 | M15.2 | 0.004 |
| Mx1 | Ghs6 | Ghs13 | 0.011 |
| Mx1 | Ghs13 | M15.2 | 0.008 |
| TLR3 | Ghs6 | Ghs13 | 0.011 |
| TLR3 | Ghs6 | M5.1 | 0.035 |
| TLR3 | Ghs13 | M15.2 | 0.004 |
| IRF1 | Ghs6 | M15.2 | 0.013 |
| IRF1 | Ghs13 | M15.2 | 0.013 |
| IRF1 | M5.1 | M15.2 | 0.013 |
| STAT1 | Ghs6 | Ghs13 | 0.013 |
| STAT1 | Ghs13 | M5.1 | 0.013 |
| STAT1 | Ghs13 | M15.2 | 0.013 |

**Supplementary Table 4. P-values from Fig 3 – Expression of innate immune genes in the Fayoumi and Leghorn sublines.**
